# Electrical Connector Assembly Based on Compliant Tactile Finger with Fingernail

**DOI:** 10.3390/biomimetics10080512

**Published:** 2025-08-05

**Authors:** Wenhui Yang, Hongliang Zhao, Chengxiao He, Longhui Qin

**Affiliations:** 1 School of Mechanical Engineering, Southeast University, Nanjing 211189, China; wenhui-yang@outlook.com (W.Y.); zhaohongliang@msn.com (H.Z.); chengxiaohe1024@outlook.com (C.H.); 2 State Key Laboratory of Mechanical Transmission for Advanced Equipment, Chongqing University, Chongqing 400044, China

**Keywords:** electrical connector assembly, biomimetic finger, compliance, tactile perception, fingernail

## Abstract

Robotic assembly of electrical connectors enables the automation of high-efficiency production of electronic products. A rigid gripper is adopted as the end-effector by the majority of existing works with a force–torque sensor installed at the wrist, which suffers from very limited perception capability of the manipulated objects. Moreover, the grasping and movement actions, as well as the inconsistency between the robot base and the end-effector frame, tend to result in angular misalignment, usually leading to assembly failure. Bio-inspired by the human finger, we designed a tactile finger in this paper with three characteristics: (1) Compliance: A soft ‘skin’ layer provides passive compliance for plenty of manipulation actions, thus increasing the tolerance for alignment errors. (2) Tactile Perception: Two types of sensing elements are embedded into the soft skin to tactilely sense the involved contact status. (3) Enhanced manipulation force: A rigid fingernail is designed to enhance the manipulation force and enable potential delicate operations. Moreover, a tactile-based alignment algorithm is proposed to search for the optimal orientation angle about the *z* axis. In the application of U-disk insertion, the three characteristics are validated and a success rate of 100% is achieved, whose generalization capability is also validated through the assembly of three types of electrical connectors.

## 1. Introduction

With the soaring demands for robotic assembly in recent years, many involved skills, such as peg-in-hole, cable routing, and parts insertion, are required in plenty of advanced manufacturing [1,2,3,4]. Therein, the insertion operation plays a significant role in various tasks. However, it still remains challenging in an unstructured environment since rather accurate perception and control are required in contact-rich scenarios, especially for the assembly of electrical connectors, which is significant in automating the production of electronic products [5,6].

In the majority of existing works, robotic assembly of connectors relies on rigid grippers, often guided by external visual and force–torque (F/T) sensors. Vision-based methods were utilized to detect the connector’s location, while impedance control is employed to minimize posture errors and handle contact forces [7]. To overcome issues like visual occlusion, some approaches formulated insertion as a regression problem using combined visual and force inputs [8], or applied deep learning with RGB-D sensors for 6D pose estimation [9]. Reinforcement learning (RL) had also been explored to adapt to novel connectors [10]. However, these methods, relying on rigid grippers and external sensors, face fundamental limitations. The wrist-mounted F/T sensor provides limited, low-dimensional feedback, making it difficult to perform complex, in-hand adjustments. Furthermore, rigid grippers possess low compliance [6] and require sophisticated control strategies to avoid jamming or damaging sensitive components, especially when facing inevitable misalignments from grasping or robot calibration errors, which can involve translational errors in the range of 10–30 mm and angular deviations as large as $45^{\circ }$ [11,12].

To address the low compliance of rigid grippers, the field has increasingly turned to soft robotic grippers. These grippers offer inherent compliance, allowing their flexible bodies to passively compensate for alignment errors and conform to complex object geometries [13]. This is crucial for practical assembly, as emphasized by compliant finray-effect grippers designed for geometry variation [14]. However, the low stiffness of most soft end-effectors often results in insufficient repeatability and insertion force, hindering their application in tasks requiring precise and firm manipulation. For instance, as surveyed in [15], high-precision assembly tasks could involve clearances as small as 2.5–7.5 μm and require controlled insertion forces that reach up to 15 N. It posed a significant challenge for purely soft structures to accomplish such tasks without buckling or losing positional accuracy. This trade-off between compliance and manipulation force remains a central challenge, sometimes leading to specially designed, non-generalizable end-effectors for specific tasks [16].

A promising direction to resolve this trade-off lies in hybrid rigid-soft designs that mimic the human fingertip. The human fingertip masterfully combines a soft pulp and a hard nail to achieve both compliance and forcefulness. The nail provides crucial structural support to enhance manipulation forces and facilitate fine-motor tasks. This synergy has been explored in several studies. For instance, a multi-layered bio-inspired fingertip with a rigid core and soft polymeric layers was developed, demonstrating a mechanical response similar to a human finger and significantly improved grasp stability [17]. Further investigation into the interaction between the soft pulp and hard nail revealed that the nail improves precision grasping not only by enhancing force but also by suppressing pulp deformation to form a “form closure” or geometric constraint on the object [18]. An extensive version of the fingernail, the retractable nail, had been integrated into a soft gripper, successfully enabling the grasping of objects as thin as 200 μm of flat surfaces [19]. These works collectively affirm the critical mechanical role of the nail in advanced robotic grasping.

On the other hand, to address the limited perception capability of external sensors, integrating tactile sensing directly into the end-effector is essential. Haptic perception is indispensable for humans, providing rich feedback for contact status estimation and action planning. To empower robots with similar capabilities, tactile sensors are often installed at the tip of robotic grippers [20,21,22,23]. These can range from tactile sensor arrays for blind bin picking of small screws [24] to vision-based tactile sensors like GelSight, which has been applied to the manipulation of electrical connectors [25,26,27]. When applied to property recognition, it has been shown to accurately discriminate between 16 different surfaces with an accuracy of 92.38% by automatically extracting features from tactile signals using a transfer learning approach [28].

However, a key element often under-explored is the integration of rich, localized tactile perception directly within this hybrid structure to guide dynamic, contact-rich tasks. How to leverage the signals from integrated sensors to fully exploit the mechanical advantages of a nail-equipped finger for a complex, closed-loop task like connector alignment remains an open question.

**Figure 1:**
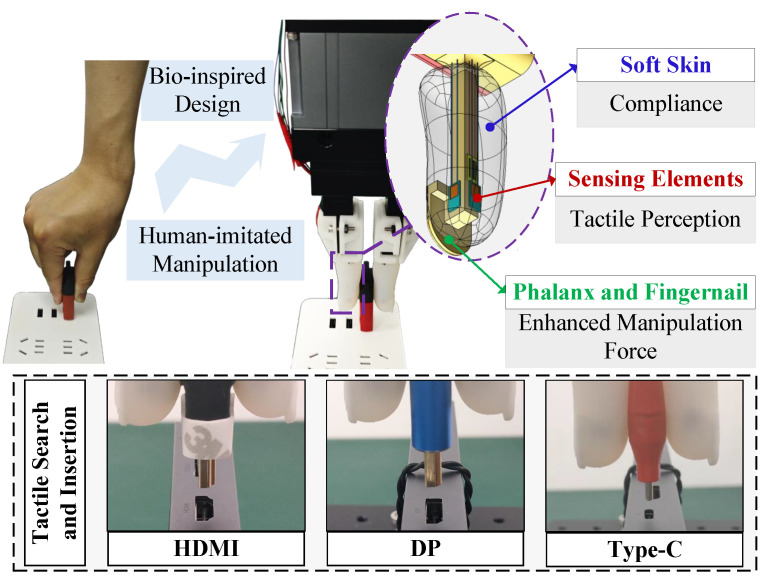
Our designed compliant tactile finger provides compliance, tactile perception, and enhanced manipulation force. The proposed tactile search strategy is successfully applied to three types of electrical connectors.

In this paper, we bridge this gap by proposing a compliant tactile finger, as shown in Figure 1, to implement the assembly of electrical connectors, which is designed mimicking the appearance, structure, and functionality of human finger and integrates tactile perception and manipulation together. It is equipped with three characteristics: (1) Compliance: A soft skin layer, as the outermost shell not only protects internal sensors but provides compliance in assembly tasks. (2) Tactile perception: Two types of sensing elements (SEs) are embedded into the soft layer (four SEs in total), which are able to perceive both dynamic and static forces. (3) Enhanced manipulation force: Considering most soft grippers suffer from insufficient manipulation force, a thin-wall structure of rigid fingernail is designed, which helps enhance the insertion and gripping force, and meanwhile increases the tactile perception sensitivity. Then, an angular alignment strategy is proposed to tackle the alignment problem between the connector and socket purely relying on tactile perception. To verify its performance, a series of robotic experiments is conducted to explore its manipulation force, compliance, and tactile perception in the application of U-disk insertion, as its sliding lid helps judge whether a complete insertion is implemented. Finally, it is translated to the other three types of electrical connectors, HDMI, DP, and Type-C. A success rate of 100% is achieved in these assembly tasks.

The main contributions of our work are as follows:

- A rigid-soft-hybrid tactile finger with a nail is designed to provide compliance, tactile perception, and enhanced manipulation force in robotic assembly.
- An angular alignment strategy is proposed, consisting of rough and fine search periods purely relying on the multi-channel tactile signals.
- A series of manipulation and assembly experiments are conducted to demonstrate the performance of our design and alignment strategy in U-disk insertion and three electrical connectors assembly.

## 2. Design and Fabrication

### 2.1. Design of the Tactile Finger

**Figure 2:**
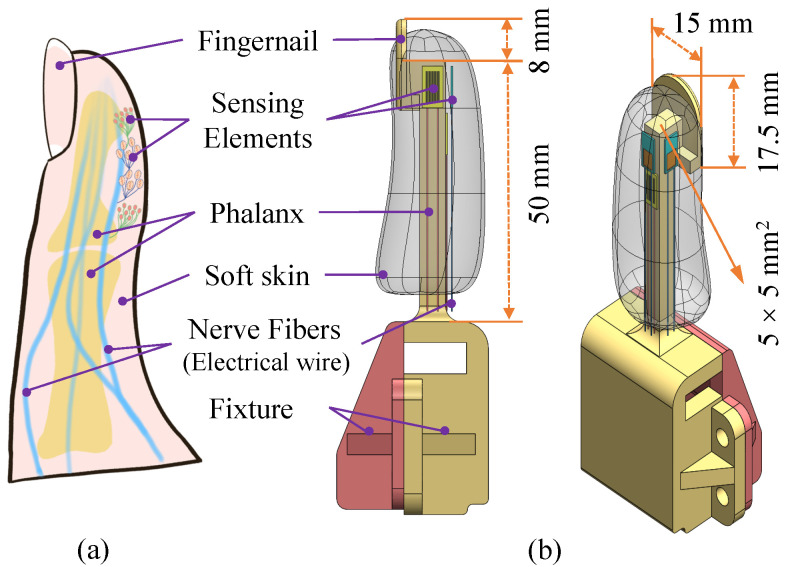
Bio-inspired design of the tactile finger. (**a**) Structure of human finger. (**b**) Structural illustration of our designed tactile finger.

Bio-inspired by the human finger, the tactile finger was designed to fully mimic it in appearance, structure, and functionality. The whole structure mainly consists of the finger part and fixture part, with which it can be conveniently installed on a robotic arm or gripper. As shown in Figure 2, a rigid PMMA (Polymethyl Methacrylate) bar (dimension: 50 × 5 × 5 mm^3^) is located at the center, playing the role of phalanx, while one end is fixed with the fixture and the other end is connected with the fingernail. Especially, the nail is a thin plate of 15 mm in width and 17.5 mm in height, sharing a similar location and size to a human fingernail. All these structures are rigid, and the phalange is surrounded by soft skin, i.e., a PDMS (Polydimethylsiloxane) layer. Four SEs are embedded into the soft skin, playing the role of mechanoreceptors within the human hand. Their produced signals are transmitted via the electronic wires, like the nerve fibers that transmit electrical impulses.

The uniqueness of this design lies in its force perception–transmission coupling mechanism, which fully utilizes the soft–rigid hybrid structure and goes beyond simple bio-mimicry of human fingers. The mechanical synergy mainly serves two purposes: (1) Enhanced Manipulation Force: The rigid fingernail acts as a crucial backing support, preventing excessive, unstructured deformation of the soft skin under high insertion loads. In combination with the rigid phalanx and soft skin, it allows the finger to withstand and transmit significantly higher axial forces, which is essential in providing sufficient mating forces for various connectors. (2) Focused Tactile Perception: With the direction orienting the fingernail constrained, a larger degree of deformations of the soft skin in the other directions is the result and, accordingly, a more significant change will be produced on the embedded SEs. As for the SGs that are attached to the rigid phalanx, deformation of the soft layer can be directly transmitted to them. As for the other type of SEs, dynamic vibration signals are conveyed to the PVDFs more easily as their ambient space is full of soft material. The coupling mechanism of rigid and soft structures, which imitates human fingers, enables robust robotic manipulation and sensitive tactile sensing.

### 2.2. Sensing Elements and Layouts

**Figure 3:**
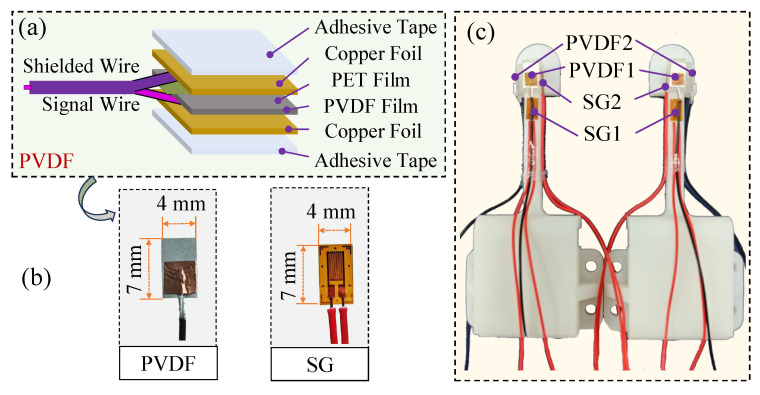
Tactile sensing elements and their arrangement on the finger skeleton: (**a**) Structure of the PVDF SE. (**b**) Fabricated PVDF and SG. (**c**) Arrangement of the four SEs on the finger skeleton.

Underneath the hand skin, there are two types of mechanoreceptors: fast-adapting (FA) and slow-adapting (SA), which are, respectively, responsible for the perception of dynamic and static stimuli. In our design, two PVDF (Polyvinylidene Fluoride) films (Figure 3a), which are piezo-electric type, are fabricated to function as the FA-I and FA-II type of mechanoreceptors. The PVDF sensor is a silver-coated film with a thickness of 52 μm and features a piezoelectric coefficient ($d_{31}$) of approximately 17 pC/N, making it highly sensitive to dynamic pressure changes. Two SG (strain gauge) films (Figure 3b) that belong to the piezo-resistive type are employed to sense the static forces as the SA-type mechanoreceptors in human skin do. The resistance of these SGs is 120 Ω and the gauge factor (K) is 2.0±0.01. All the SEs share the same dimension of 7 × 4 mm^2^. Thus, both dynamic and static forces can be perceived with the tactile finger.

In order to sense stimuli from different directions, the PVDF and SG SEs are arranged perpendicular to each other within each type. One is parallel to the fingernail while the other is perpendicular to it. Considering that PVDF is sensitive to dynamic stimuli, its surrounding space is filled with soft material, which conduces to the spread of vibration signals. In contrast, the SG is attached to the rigid phalange and also surrounded by soft skin, which not only eases the fabrication of the tactile finger but also increases the deformation degree.

### 2.3. Fabrication of the Humanoid Finger and Tactile Gripper

Another advantage of the tactile finger is that it is easy to fabricate, which mainly includes five steps: (1) Preparation: The finger skeleton, i.e., the phalanx, fixture, and fingernail, needs to be manufactured via 3D printing in advance. Meanwhile, a mold that accords with our designed finger’s appearance should be manufactured. (2) Fabrication of SEs: PVDFs and SGs are fabricated with the electrodes and electronic wires connected. (3) Attachment of SEs. PVDFs are attached to a small soft PDMS cube that is fabricated in advance via curing, while the SGs are pasted to the phalanx directly. (4) PDMS liquid is obtained by mixing its two components, material and degassed, via a vacuum pump to remove possible bubbles. (5) The PDMS mixture is poured into the curing setup. To ensure the consistency between the tactile finger and gripper, the fixture is installed on the gripper in advance. Finally, a tactile gripper is obtained after 6 h of curing.

## 3. Angular Alignment Strategy

### 3.1. U-Disk Insertion Problem

U-disk insertion is selected among various peg-in-hole applications on account of two considerations: (1) The USB connector is widely used in the assembly of most electronic devices. (2) Different from the typical cylinder–hole assembly, U-disk contains more complex geometry and structures, e.g., multiple pins and mating surfaces. Moreover, a large enough insertion force is required for a complete assembly, while the involved force varies highly nonlinearly during the whole insertion process.

**Figure 4:**
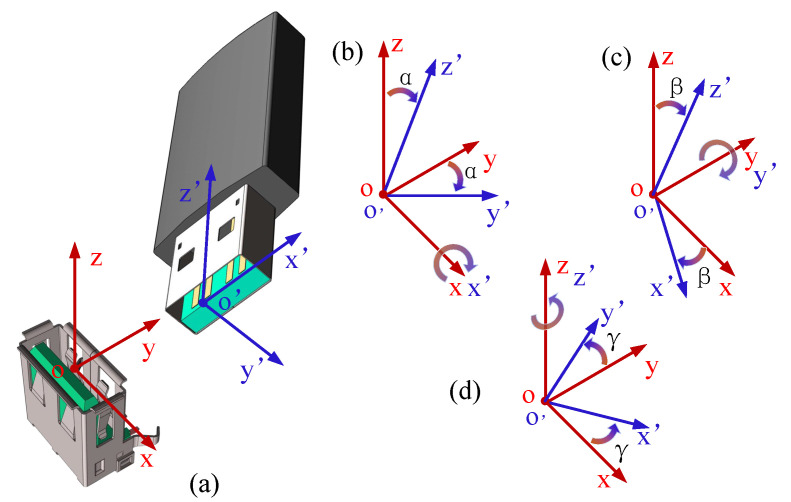
Problem description of U-disk insertion: (**a**) U-disk and socket with their attached coordinate frame *o*-xyz and $o^{\prime }$-$x^{\prime }y^{\prime }z^{\prime }$. (**b**–**d**) Three orientation deviations, respectively, rotating α, β, γ angles about x, y, z axes.

As shown in Figure 4a, the socket is fixed with its coordinate frame, defined as *o*-xyz, where the origin point is located at the center of its uppermost plane. The U-disk is gripped with our developed tactile gripper, and it is accompanied by a local moving frame $o^{\prime }$-$x^{\prime }y^{\prime }z^{\prime }$. A complete assembly pipeline usually consists of grasping, approaching, positioning, alignment, execution, etc. Therein, the approaching and positioning procedures can be realized by visual-servo methods [29], which will guide the gripper to a relatively accurate position. However, the grasping action and the calibration error between *o*-xyz and $o^{\prime }$-$x^{\prime }y^{\prime }z^{\prime }$ easily lead to their angular misalignment, which significantly affects the assembly result. Therefore, we limit ourselves to focusing on the angular alignment between the U-disk and the socket in this paper. Via the application of U-disk insertion, the passive compliance of our soft–rigid hybrid gripper will be verified through the two occasions as given in Figure 4b,c, where the insertion is implemented under different angular uncertainties: angle α about *x* axis, and angle β about *y* axis. The angular tolerance will be found out with the experimental explorations on compliance in Section 4.

Besides, due to the occlusion phenomenon, visual guidance is difficult to ensure precise alignment of angle γ, which, however, is critical for the insertion task. How to find out the optimal orientation angle γ of the gripper frame $o^{\prime }$-$x^{\prime }y^{\prime }z^{\prime }$ relative to the target socket frame *o*-xyz is tackled in this paper.

### 3.2. Tactile-Based Alignment Algorithm

Bio-inspired by the search strategy of human operators in a dark environment, we propose an active exploration method to blindly find the optimal rotation angle $\tilde{\gamma }$ purely relying on tactile signals. The alignment procedure in the connector assembly involves both positional and orientational alignment. Therein, the positional alignment has been investigated by plenty of works, and the vision-guided manner is widely adopted [30,31]. In this work, we mainly focus on the orientational alignment solely relying on tactile perception. Therefore, the position is fixed as a priori information in all experiments, with only the orientation angle varying. The compliance of the tactile finger allows for contact exploration between the U-disk and the socket. When the finger contacts the socket with an incorrect orientation angle, an increased contact force is expected, which recovers to a minimum when the U-disk and the socket hole are aligned (at $0^{\circ }$ or $180^{\circ }$).

The searching algorithm is detailed in Algorithm A1 in the Appendix A and consists of a rough search followed by a fine search. The specific peak detection process is implemented as follows:

- Rough Search: The algorithm first identifies the time instant $t_{\min}$ where the static force deviation is minimal. This is achieved by calculating the absolute difference between the current SG1 signal value $S_{3}\left(t\right)$ and its initial value at the start of the sweeping process $S_{3}\left(t_{\mathrm{start}}\right)$ and then finding the time that minimizes this difference. This instant $t_{\min}$ indicates the approximate moment of best alignment where the static pressure is closest to the non-contact state.
- Fine Search: A search window $T=[t_{\min}-t_{dmax},t_{\min}]$ is established, where $t_{dmax}$ is the maximal expected time delay. Within this window, the algorithm locates the dynamic event markers by finding the time instants that correspond to the maximum absolute values of the PVDF1 and PVDF2 signals ($|S_{1}\left(t\right)|$ and $|S_{2}\left(t\right)|$). The instants $t_{\mathrm{peak}1}$ and $t_{\mathrm{peak}2}$ represent the moments of most significant dynamic change.

The final optimal time, $t_{\mathrm{optimal}}$, is then calculated by averaging $t_{\mathrm{peak}1}$ and $t_{\mathrm{peak}2}$, from which the optimal angle ${\tilde{\gamma }}_{\mathrm{optimal}}$ is derived. This two-stage approach leverages the stability of the static sensor for initial guidance and the sensitivity of the dynamic sensors for final precision.

## 4. Experimental Design

### 4.1. Experimental Setup

**Figure 5:**
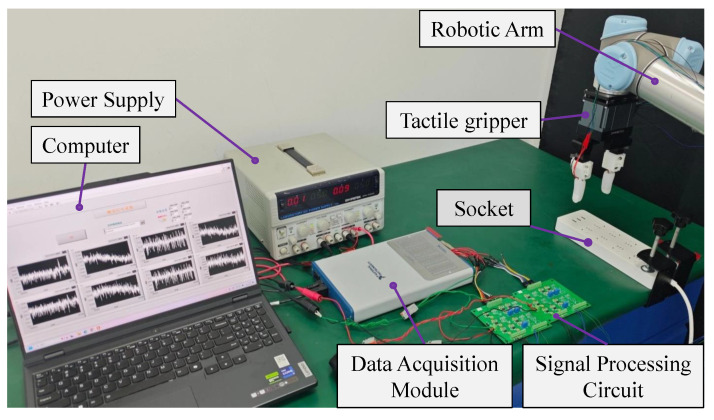
The experimental platform for robotic manipulation.

An experimental setup for robotic manipulation is established to test the assembly and perception performance. The same tactile finger was utilized across all experiments to ensure the consistency of the performance evaluation. The experiment was conducted in a normal environment without any environmental factors being controlled. Although the SG SE would be affected by the temperature change, no significant changes would occur during the whole experiment. Moreover, the surrounding soft skin layer has poor thermal conductivity, further alleviating the influence of temperature changes. The other factors, such as humidity and light, have no impact on the performance. As shown in Figure 5, the tactile gripper is installed on a robotic arm (UR5, Universal Robot Inc., Odense, Denmark), which is implemented in an ROS (Robot Operating System) environment. The produced tactile signals pass through a signal processing circuit and then are acquired by a computer via a data acquisition module (USB6363, National Instruments Corp., Austin, TX, USA). In the acquisition of tactile signals, electromagnetic interference in the ambient environment should be taken into consideration. In this work, a band-stop filter was designed to lower the influence of the electronic noise in the pre-processing stage.

### 4.2. Comparative Experiment of Insertion Force

**Figure 6:**
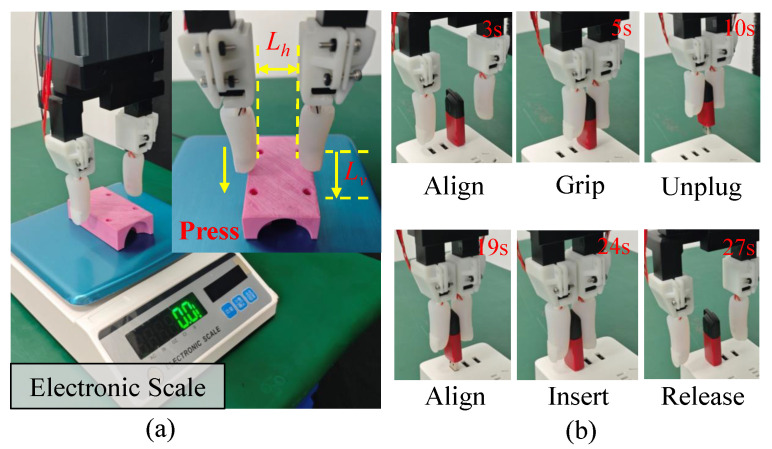
(**a**) Setup for insertion force measurement, $L_{v}=5$ mm, $L_{h}=28$ mm. (**b**) Procedures of the U-disk unplugging and insertion task.

To verify the insertion force of our proposed tactile finger, two comparative experiments are conducted: (1) Insertion force measurement (Figure 6a). (2) U-disk unplugging and insertion (Figure 6b). A reference tactile finger is prepared with the fingernail removed to verify the role of the fingernail in increasing the insertion force.

In comparative experiment (1), the tactile gripper is first positioned vertically above a rigid part on an electronic scale and closed to a fingertip distance of $L_{h}=28$ mm. The gripper is then lowered until its fingertips make initial contact with the part. Subsequently, it moves downwards for a pre-defined vertical distance of $L_{v}=5$ mm over a duration of $T_{p}=10$ s to press on the part. After holding the position for $T_{s}=3$ s, the gripper is lifted up. The setup, as shown in the inset of Figure 6a, ensures that only the soft region of the fingertip makes contact.

In comparative experiment (2), the tactile gripper is adjusted and aligned to the rightmost USB port from 0 s to 3 s. Then, it closes to grasp the U-disk during 3 s∼5 s. Subsequently, the gripper unplugs the U-disk during 5 s∼10 s, transfers it to the target USB port, and aligns it in 10 s∼19 s. Finally, the gripper moves downwards for a distance of $D^{*}=15$ mm over a duration of $T^{*}=5$ s to complete the insertion. After the insertion is finished, the gripper releases the U-disk at 24 s.

### 4.3. Compliance Verification of U-Disk Insertion

Compliance of the tactile gripper is verified via a U-disk insertion with the α and β angles in Figure 4b,c varying. Specifically, at the beginning of each insertion task, uncertainty is imposed on the orientation angles artificially. Then the insertion action is implemented along the planned path. The angle deviations for α and β include $dev\left(\alpha \right)=\left[0,-3^{\circ },3^{\circ },-4^{\circ },4^{\circ },-5^{\circ },5^{\circ }\right]$, $dev\left(\beta \right)=\left[0,-3^{\circ },3^{\circ },-4^{\circ },4^{\circ },-5^{\circ },5^{\circ },6^{\circ },-6^{\circ }\right]$. The experiment will be repeated 10 times in each situation to obtain the success rate.

### 4.4. Angular Alignment in U-Disk Insertion and Electrical Connectors Assembly

The robot is controlled to implement the procedures in Algorithm A1, aimed at finding out the optimal angular orientation angle ${\tilde{\gamma }}_{optimal}$. The lowest height of the fingertip is $z_{min}^{\prime }=-1$ mm, meaning the finger continues to lower for 1 mm after contact with the socket. The lower and upper bounds for tactile search are set as $\gamma_{lb}=-90^{\circ }$ and $\gamma_{ub}=90^{\circ }$. The sweeping process is fast, only lasting 3 s in total. Once the optimal angle is determined, the U-disk insertion task will be implemented with the found parameters.

Then, the blind tactile search algorithm is applied to the assembly of three other types of electrical connectors: HDMI, DP, and Type-C, to further verify its generalization capability. Similarly, the assembly is implemented once the optimal orientation angle ${\tilde{\gamma }}_{optimal}$ is found. All these assembly tasks will be repeated 10 times as well.

## 5. Results

### 5.1. Insertion Force

**Figure 7:**
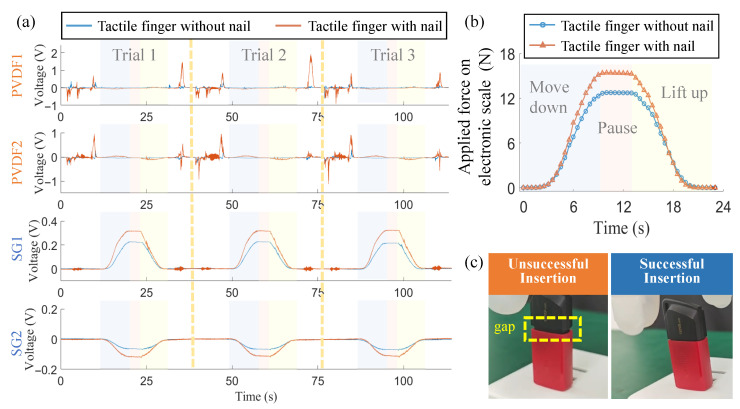
(**a**) Tactile signals from the four SEs during the comparative experiment (2). (**b**) Force measured by the electronic scale. (**c**) Insertion result using the tactile finger with and without fingernail.

Based on the experimental setup in Figure 6a, we obtain the pressure force from the electronic scale by converting the measured mass into force via F=mg and g=9.8 m/s^2^. As indicated in Figure 7a,b, the whole experiment consists of three processes: moving down, pausing, and lifting up. In the first process, the tactile gripper moves downwards vertically, and the pressure begins to increase from 2 s, indicating the fingertip starts to contact with the part on the scale. Then the pressure force continues to increase when the gripper is still lowered, and the deformation of the tactile finger increases. At 10 s, the gripper stops moving and the maximal pressure force reaches 15.4 N for our designed humanoid finger with nail. After a 3 s pause, the gripper starts to lift up and, correspondingly, the pressure force decreases. At 21 s, the gripper loses contact with the part when the pressure force recovers to 0 N. In contrast, the maximal pressure force produced by the tactile finger without a nail is 12.8 N. It is found that the added nail structure can improve the axial pressure force by 20.3%. More importantly, it should be noted that the pressure force is produced by the soft fingertip region since the fingernail does not contact the rigid part throughout the whole process. It indicates the fingernail contributes to changing the deformation of the soft skin. This phenomenon occurs because the rigid nail acts as a backing support, suppressing the excessive deformation of the soft pulp. This suppression allows the fingertip to withstand greater compressive forces before yielding. As validated in [18], this interaction between the hard nail and soft pulp can also lead to a “form closure” effect, where the skin conforms more tightly to the object, effectively forming a geometric constraint that enhances the overall force output.

Tactile signals in the pressure process are given in Figure 7a. It should be noted that PVDF1 and SG1 are parallel to the nail and closer to the contact area. Therefore, the amplitudes of PVDF1 and SG1 are found to be evidently larger than those of PVDF2 and SG2. In general, the PVDFs, acting as dynamic sensors, primarily respond to transient events. The signal peaks observed in the non-contact phases of the trials correspond to vibrations caused by the acceleration and deceleration of the robotic arm, while their response during the slow, quasi-static pressing phase is minimal. In contrast, the SGs respond to static forces. The voltage output from the SGs is converted from the resistance change via a Wheatstone bridge, where the polarity (positive or negative voltage) indicates the direction of bending. The SG1 signal closely follows the variation of the actual forces shown in Figure 7b. The SG2 signal, being mounted on the side of the phalanx, shows an opposite trend. This negative voltage suggests that as the fingertip is compressed axially, the phalanx experiences a slight bending or strain in a direction opposite to that of SG1, likely due to the complex deformation of the surrounding soft skin. In addition, the experiment is repeated three times to verify its repeatability of the tactile perception. Both PVDF and SG signals are found to have strong repeatability throughout the three trials.

When the tactile finger with and without a fingernail is utilized to implement the U-disk insertion task, the result is given in Figure 7c. Although both fingers can insert the U-disk into the socket, the insertion without fingernail involvement is not completely successful since there still exists a gap between the sliding lid and the U-disk head. It also proves that the fingernail structure is helpful in providing larger manipulation forces.

### 5.2. Compliance Result in U-Disk Insertion

To test its compliance in U-disk insertion, we control the robot to adjust the orientation angle of the gripper. At first, the orientation angle α, i.e., the rotation angle about *x* axis between o−xyz frame and $o^{\prime }-x^{\prime }y^{\prime }z^{\prime }$ frame, is changed. The test cases include $\alpha =[0^{\circ },-3^{\circ },3^{\circ },-4^{\circ },4^{\circ },-5^{\circ },5^{\circ }]$. Therein, the U-disk is not completely inserted in cases $\alpha =-5^{\circ }$ and $5^{\circ }$, as there is a gap between the sliding lid and the U-disk head. In other cases, the insertion tasks are implemented completely, where self-righting is observed due to the passive compliance. The allowed angle range of α is $\left[-4^{\circ },4^{\circ }\right]$ for a complete successful insertion. The specific result can be found in Table 1.

**Figure 8:**
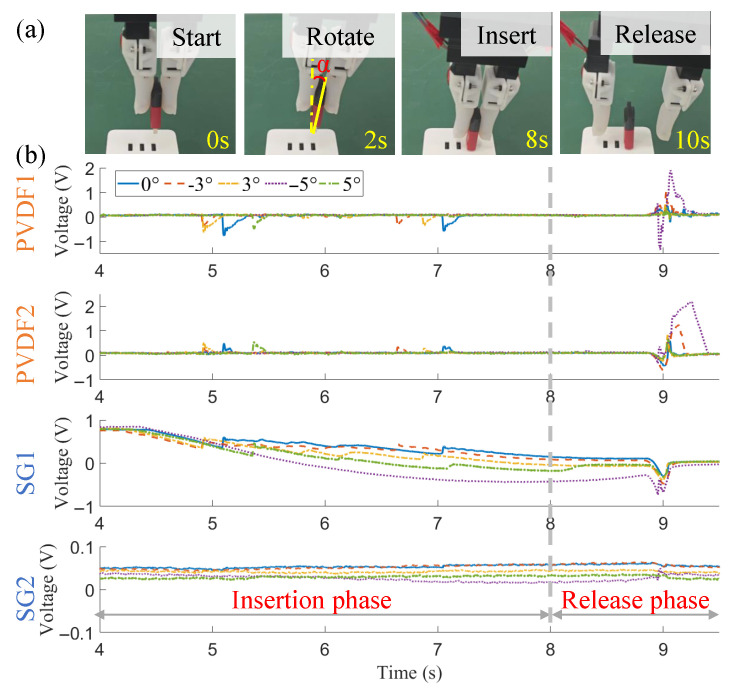
Snapshots (**a**) and tactile signals (**b**) for compliance verification with orientation angle α ranging in $\left[-5^{\circ },5^{\circ }\right]$.

In Figure 8b, tactile signals during the insertion process are given. In general, significant variations of PVDF signals are found only in the onset of contact, sliding, and release, and other dynamic instants. No apparent changes are found from SG2. In contrast, the signal from SG1 shows obvious variation corresponding to different insertion phases. As for different orientation angles α, the time instants at which signal peaks are generated at PVDFs and SG1 are found to be distinctly different. It provides a possibility for us to infer the current deviation directions and further, make an adjustment for finer alignment of α.

**Figure 9:**
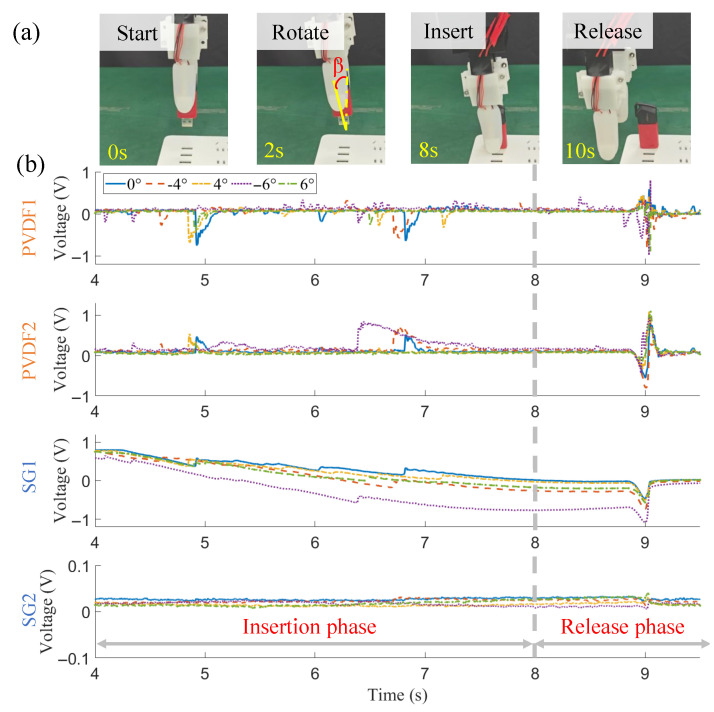
Snapshots (**a**) and tactile signals (**b**) for compliance verification with orientation angle β ranging in $\left[-6^{\circ },6^{\circ }\right]$.

Then, the orientation angle β, i.e., the rotation angle about *y* axis between *o*-xyz frame and $o^{\prime }$-$x^{\prime }y^{\prime }z^{\prime }$ frame, is changed. The test cases include $\beta =[0^{\circ },-3^{\circ },3^{\circ },-4^{\circ },4^{\circ },-5^{\circ },5^{\circ },-6^{\circ },6^{\circ }]$. The insertion can be implemented successfully within $\left[-5^{\circ },5^{\circ }\right]$, while exceeding this range cannot ensure a completely successful insertion. The specific result is given in Table 2. Its allowed angle range of β is $\left[-5^{\circ },5^{\circ }\right]$, a little larger than the range of α. Tactile signals during this process are given by Figure 9b, in which a very similar conclusion to the case of α can be drawn. The only difference is that when the angular deviation is larger, more dynamic vibrations are observed in the PVDF signals.

### 5.3. Angular Alignment

**Figure 10:**
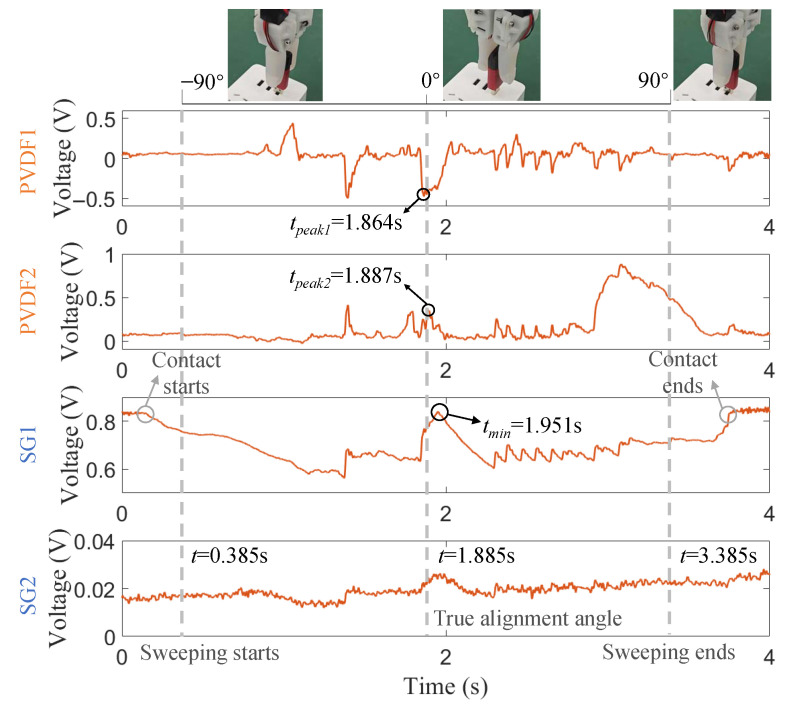
Tactile signals from the searching process for optimal angular alignment.

According to Algorithm A1, the tactile gripper implements a press-and-sweeping action quickly. The sweeping time is set as 3 s in this paper, which is found nearly no influence on the final orientation result. As shown in Figure 10, the lower and upper bounds for the optimal angle ${\tilde{\gamma }}_{optimal}$ are, respectively, $-90^{\circ }$ and $90^{\circ }$. The expected true orientation angle is $0^{\circ }$.

Through the four channels of SEs, the static force of SG1 still shows the most apparent variation and close relations to the status in different phases. SG2 appears to have a very similar trend, but with relatively tiny signal fluctuations. From the contact start point on, the perceived force begins to decrease, corresponding to the press action. Then, the sweeping process starts, accompanied by a different downtrend. When the current orientation angle γ approximates the true angles ($0^{\circ }$, t=1.885 s), a rising peak can be found, and the amplitude nearly equals the non-contact level at the beginning. It is because the U-disk stays in the right orientation at the current instant, and no pressure force is applied to it. The corresponding time instant is $t_{min}=1.951$. However, a remarkable time delay, i.e., $t_{delay}=0.066$ s, exists between the true time instant and the found $t_{min}$. It accords with the expectation as a short time is required for the deformation recovery of soft skin when applied forces are released.

In Algorithm 1, $td_{max}=0.15$ s and then the fine search is implemented within t∈ [1.801 s, 1.951 s]. It can be found that the variation trend between PVDF1 and PVDF2 is opposite for dynamic stimuli. Therefore, the lowest peak of PVDF1 and the highest peak of PVDF2 are, respectively, found: $t_{peak1}=1.864$ s and $t_{peak2}=1.887$ s. As the sweeping process is implemented at uniform angular velocity, the estimated optimal orientation angle can be easily calculated as ${\tilde{\gamma }}_{optimal}=-0.57^{\circ }$. Within this angular deviation, the U-disk insertion task is accomplished successfully.

The blind tactile search and U-disk insertion are repeated 10 times. A success rate of 100% is achieved as shown in Table 3 and its maximal error of estimated orientation angle, $e_{\gamma ,max}$, is $-1.44^{\circ }$. When applied to the assembly of the other three types of electrical connectors, i.e., HDMI, DP, and Type-C, the maximal errors are $-1.47^{\circ }$, $-1.44^{\circ }$, and $-1.29^{\circ }$, respectively. All the assembly tasks are implemented successfully even under such misalignment, which is inferred by benefiting from the compliance of the tactile finger.

## 6. Conclusions

A compliant tactile finger was designed in this paper for electronic connectors assembly. Bio-inspired by human fingers, the tactile finger fully mimicked its appearance, structure, and functionality. Compared with a traditional rigid gripper, it was equipped with three characteristics: compliance, tactile perception, and enhanced insertion force. Meanwhile, a tactile-based angular alignment algorithm was proposed to actively find out the optimal orientation angle about the z-axis. A series of experiments were conducted to comparatively validate its performance. Finally, a U-disk and three types of electrical connectors are assembled successfully, from which the tactile perception has shown significant potential in robotic assembly.

The main contribution of this work lies in the development of a novel tactile-based alignment algorithm that actively finds the optimal orientation for insertion, purely relying on multi-channel tactile data. This strategy eliminates the need for external vision in occluded scenarios. The effectiveness and generalization capability of our integrated approach were validated by achieving a 100% success rate in the assembly of four different types of electrical connectors (U-disk, HDMI, DP, and Type-C), even under initial misalignments. This demonstrates that a holistic solution, coupling bio-inspired mechanics with intelligent tactile perception, can robustly solve complex, contact-rich industrial tasks. While the results are promising, the current algorithm primarily addresses single-axis angular alignment.

While our proposed tactile-based alignment method primarily relies on the geometrical mating of a pair of connectors, it still suffers from several limitations and needs further investigation in the future. For example, the method is valid when the changes in contact status between two connector parts can be detected. Thus, it is applicable to most existing electrical connectors, whether symmetrical or asymmetrical types. However, it may fail in some centrosymmetric geometries, such as those with round connectors that exhibit no orientation-related variations. Meanwhile, only four types of electrical connectors were employed for verification in this work, which is insufficient to ensure a more practical and general application of our tactile finger. In the future, plenty of different connectors should be added, covering more sizes, geometries, and complexities. In practical application of the tactile finger, there are some other issues to consider. For instance, the aging and wear of the soft skin may affect the compliance and perception performance after long-term applications. What is more, the impact of the properties of the fingernail on the performance is not addressed in this work. These problems can be further explored as promising directions to fill the gap between finger verification and practical applications. Beyond these points, our primary future work will focus on extending this strategy to handle full 6-DOF pose uncertainties, potentially by leveraging machine learning techniques for more sophisticated tactile signal processing.

## Figures and Tables

**Table 1:** Compliance result of rotation angle α.

α	$-5^{\circ }$	$-4^{\circ }$	$-3^{\circ }$	0^∘^	3^∘^	4^∘^	5^∘^
**success rate**	0/10	10/10	10/10	10/10	10/10	10/10	0/10

**Table 2:** Compliance result of rotation angle β.

β	$-6^{\circ }$	$-5^{\circ }$	$-4^{\circ }$	$-3^{\circ }$	0^∘^	3^∘^	4^∘^	5^∘^	6^∘^
**success rate**	1/10	10/10	10/10	10/10	10/10	10/10	10/10	9/10	0/10

**Table 3:** Assembly result of the U-disk and other 3 types of electrical connectors with the angular alignment strategy.

**U-disk**	success rate	$e_{\gamma ,max}$	**HDMI**	success rate	$e_{\gamma ,max}$
	10/10	$-1.44^{\circ }$		10/10	$-1.47^{\circ }$
**DP**	success rate	$e_{\gamma ,max}$	**Type-C**	success rate	$e_{\gamma ,max}$
	10/10	$-1.44^{\circ }$		10/10	$-1.29^{\circ }$

## Data Availability

The data presented in this study are available on request from the corresponding author.

## Appendix A. Tactile-Based Alignment Algorithm

**Algorithm A1:** The Tactile-based Alignment Algorithm

## References

[1] Van Wyk K., Culleton M., Falco J., Kelly K. (2018). Comparative peg-in-hole testing of a force-based manipulation controlled robotic hand. IEEE Trans. Robot..

[2] Waltersson G.A., Laezza R., Karayiannidis Y. (2022). Planning and control for cable-routing with dual-arm robot. Proceedings of the 2022 International Conference on Robotics and Automation (ICRA).

[3] Tang T., Lin H.C., Zhao Y., Fan Y., Chen W., Tomizuka M. (2016). Teach industrial robots peg-hole-insertion by human demonstration. Proceedings of the 2016 IEEE International Conference on Advanced Intelligent Mechatronics (AIM).

[4] Liu J., Liu J., Zhang Z., Xu J., Lin H. (2016). Anytime RRT based cable automatic routing under three-dimensional environment. Jixie Gongcheng Xuebao/Chin. J. Mech. Eng..

[5] Jiang Y., Huang Z., Yang B., Yang W. (2022). A review of robotic assembly strategies for the full operation procedure: Planning, execution and evaluation. Robot. Comput.-Integr. Manuf..

[6] Yumbla F., Yi J.S., Abayebas M., Shafiyev M., Moon H. (2020). Tolerance dataset: Mating process of plug-in cable connectors for wire harness assembly tasks. Intell. Serv. Robot..

[7] Song H.C., Kim Y.L., Lee D.H., Song J.B. (2017). Electric connector assembly based on vision and impedance control using cable connector-feeding system. J. Mech. Sci. Technol..

[8] Spector O., Di Castro D. (2021). Insertionnet-a scalable solution for insertion. IEEE Robot. Autom. Lett..

[9] Zhang K., Wang C., Chen H., Pan J., Wang M.Y., Zhang W. (2023). Vision-based six-dimensional peg-in-hole for practical connector insertion. Proceedings of the 2023 IEEE International Conference on Robotics and Automation (ICRA).

[10] Nair A., Zhu B., Narayanan G., Solowjow E., Levine S. (2023). Learning on the job: Self-rewarding offline-to-online finetuning for industrial insertion of novel connectors from vision. Proceedings of the 2023 IEEE International Conference on Robotics and Automation (ICRA).

[11] Wang Z., Li X., Zhao H., Shao L., Ma X., Zou H., Ding H. (2024). Geometry and Force Guided Robotic Assembly With Large Initial Deviations for Electrical Connectors. IEEE Trans. Autom. Sci. Eng..

[12] Yun J.W., Na M., Hwang Y., Song J.B. (2025). Similar assembly state discriminator for reinforcement learning-based robotic connector assembly. Robot. Comput.-Integr. Manuf..

[13] Shintake J., Cacucciolo V., Floreano D., Shea H. (2018). Soft robotic grippers. Adv. Mater..

[14] Hartisch R.M., Haninger K. (2023). Compliant finray-effect gripper for high-speed robotic assembly of electrical components. Proceedings of the 2023 IEEE/ASME International Conference on Advanced Intelligent Mechatronics (AIM).

[15] Li R., Qiao H. (2019). A survey of methods and strategies for high-precision robotic grasping and assembly tasks—Some new trends. IEEE/ASME Trans. Mechatronics.

[16] Yumbla F., Abayebas M., Yi J.S., Jeon J., Moon H. (2021). Reposition and alignment of cable connectors using a vibration plate manipulator for wire harness assembly tasks. Int. J. Precis. Eng. Manuf..

[17] Controzzi M., D’ Alonzo M., Peccia C., Oddo C.M., Carrozza M.C., Cipriani C. (2014). Bioinspired fingertip for anthropomorphic robotic hands. Appl. Bionics Biomech..

[18] Kumagai A., Obata Y., Yabuki Y., Jiang Y., Yokoi H., Togo S. (2023). Improvement of precision grasping performance by interaction between soft finger pulp and hard nail. Soft Robot..

[19] Jain S., Stalin T., Subramaniam V., Agarwal J., Valdivia y Alvarado P. (2020). A soft gripper with retractable nails for advanced grasping and manipulation. Proceedings of the 2020 IEEE International Conference on Robotics and Automation (ICRA).

[20] Sundaralingam B., Hermans T. (2021). In-hand object-dynamics inference using tactile fingertips. IEEE Trans. Robot..

[21] Qin L., Shi X., Wang Y., Zhou Z. (2023). Perception of static and dynamic forces with a bio-inspired tactile fingertip. J. Bionic Eng..

[22] Shi X., Wang Y., Qin L. (2023). Surface Recognition with a Bio-inspired Tactile Fingertip. IEEE Sens. J..

[23] Ades C., Abd M.A., Hutchinson D.T., Tognoli E., Du E., Wei J., Engeberg E.D. (2024). Biohybrid Robotic Hand to Investigate Tactile Encoding and Sensorimotor Integration. Biomimetics.

[24] Ishige M., Umedachi T., Ijiri Y., Taniguchi T., Kawahara Y. (2020). Blind bin picking of small screws through in-finger manipulation with compliant robotic fingers. Proceedings of the 2020 IEEE/RSJ International Conference on Intelligent Robots and Systems (IROS).

[25] Caccavale R., Finzi A., Laudante G., Natale C., Pirozzi S., Villani L. (2023). Manipulation of Boltlike Fasteners Through Fingertip Tactile Perception in Robotic Assembly. IEEE/ASME Trans. Mechatronics.

[26] Li R., Platt R., Yuan W., Ten Pas A., Roscup N., Srinivasan M.A., Adelson E. (2014). Localization and manipulation of small parts using gelsight tactile sensing. Proceedings of the 2014 IEEE/RSJ International Conference on Intelligent Robots and Systems.

[27] Okumura R., Nishio N., Taniguchi T. (2022). Tactile-sensitive newtonianvae for high-accuracy industrial connector insertion. Proceedings of the 2022 IEEE/RSJ International Conference on Intelligent Robots and Systems (IROS).

[28] Qin L., Shi X., Yang W., Qin Z., Yi Z., Shen H. (2024). Surface Recognition with a Tactile Finger based on Automatic Features Transferred from Deep Learning. IEEE Trans. Instrum. Meas..

[29] Ma Y., Liu X., Zhang J., Xu D., Zhang D., Wu W. (2020). Robotic grasping and alignment for small size components assembly based on visual servoing. Int. J. Adv. Manuf. Technol..

[30] Xu R., Zhao X., Liu F., Tao B. (2023). High-precision monocular vision guided robotic assembly based on local pose invariance. IEEE Trans. Instrum. Meas..

[31] Wang H., Salunkhe O., Quadrini W., Lämkull D., Ore F., Despeisse M., Fumagalli L., Stahre J., Johansson B. (2024). A systematic literature review of computer vision applications in robotized wire harness assembly. Adv. Eng. Inform..

